# Efficacy of different irrigant activation techniques for cleaning root canal anastomosis

**DOI:** 10.1186/s12903-023-02835-0

**Published:** 2023-03-11

**Authors:** O. K. Montaser, D. M. Fayyad, N. Abdelsalam

**Affiliations:** Department of Endodontics, Faculty of Dentistry, Suez Canal University, Ismailia, Egypt

**Keywords:** EDDY, Final irrigation, Anastomosis, Isthmus, Passive ultrasonic irrigation

## Abstract

**Objective:**

This study compared in vitro the anastomosis cleaning efficacy of different irrigant activation techniques at different levels; control group non-activation (NA), passive ultrasonic irrigation (PUI) using Irrisafe, and EDDY sonic activation.

**Methods:**

Sixty anastomosis-containing mesial roots of mandibular molars were mounted in resin, sectioned at 2, 4, and 6 mm from the apex. Then reassembled and instrumented in a copper cube. For the irrigation technique roots were randomly divided into 3 groups (*n* = 20): group 1: NA, group 2: Irrisafe, group 3: EDDY. Stereomicroscopic images of anastomoses were taken after instrumentation and after irrigant activation. ImageJ program was used to calculate the percentage of anastomosis cleanliness. The percentage of cleanliness was calculated before and after final irrigation within each group and were then compared using paired t-tests. Intergroup and intragroup analyses were performed to compare between different activation techniques at the same root canal level (2, 4 and 6 mm) (intergroup) and to evaluate if each technique had different cleanliness efficacy according to the root canal level (intragroup) using one-way analysis of variance and post hoc tests (*p* < 0.05).

**Results:**

All three irrigation techniques significantly improved anastomosis cleanliness (*p* < 0.001). Both activation techniques were significantly better than the control group at all levels. Intergroup comparison revealed that EDDY significantly achieved the best overall anastomosis cleanliness. The difference between EDDY and Irrisafe was significant in favor to EDDY at 2 mm and insignificant at 4 and 6 mm. The intragroup comparison showed that improvement in anastomosis cleanliness (i2-i1) in the needle irrigation without activation group (NA) was significantly higher in the apical 2 mm level compared to the 4 & 6 levels. While the difference in anastomosis cleanliness improvement (i2-i1) between levels in both Irrisafe and EDDY groups was insignificant.

**Conclusions:**

Irrigant activation improves anastomosis cleanliness. EDDY was the most efficient in cleaning anastomoses located in the critical apical part of the root canal.

**Clinical relevance:**

Cleaning and disinfection of the root canal system followed by apical and coronal sealing is the key for healing or prevention of apical periodontitis. Remnants of debris and microorganisms retained within the anastomoses (isthmuses), or other root canal irregularities may lead to persistent apical periodontitis. Proper irrigation and activation are essential for cleaning root canal anastomoses.

## Introduction

The ultimate goal of root canal therapy is to prevent apical periodontitis or to allow healing of an existing-apical periodontitis by rendering the root canal system as free as possible from microorganisms. This is accomplished by disinfecting the whole root canal system through meticulous shaping and irrigation [1].

Most often clinicians deal with narrow curved roots in which there is more than one canal, those canals are usually connected through fins or anastomoses. An anastomosis -also called an isthmus- is a narrow space between two root canals that can harbor microorganisms and dentin debris packed into it during instrumentation [2], Clinical observations showed that persistent apical periodontitis is most frequent in molar teeth with their relatively complex anatomy- specially isthmuses, fins and lateral canals-that can prevent efficient cleaning and three-dimensional disinfection of the whole root canal system [3, 4].

Sodium hypochlorite (NaOCl) is the most used irrigating solution. It has a strong antimicrobial effect with the ability to dissolve organic tissue [5]. Passive ultrasonic irrigation (PUI) was advocated for activation of NaOCl, to improve its ability to clean anatomically complex areas via cavitation and acoustic streaming [5]. Irrigant activation by sonic energy is another popular method. The EDDY tip (VDW GmbH, Munich, Germany) made of a flexible polyamide is one of the sonic activation systems. According to the manufacturer, it enables efficient root canal cleaning without the drawbacks of ultrasonic-activated systems, such as the diminished effectiveness upon touching the dentinal walls or even creating irregularities within these walls [6].

Anastomosis is a restricted area that harbors remnants of pulp tissue, bacterial biofilm and infected instrumentation debris especially in infected necrotic root canals thus it must be cleaned and disinfected to achieve a favorable outcome, it is well-agreed that the anastomosis cannot be touched by the instruments and is a very difficult area to be reached by the irrigant [7]. Therefore, many studies aimed to investigate the efficiency of different irrigation techniques to clean root canal anastomosis without concluding a specific technique that completely eliminates debris from these areas [8]. Root canal anastomosis could be present at any level from apical to coronal along the connection between the two root canals, most commonly at 2 mm [9] and 4-6 mm from the apical foramen [10]. The flow of the irrigant and subsequent cleaning of the main root canal at different root canal levels decreases from coronal to apical [11], similarly cleaning root canal anastomoses differs at various levels with different methods of irrigation. The efficiency of EDDY versus other activation techniques for anastomosis cleaning at different levels wasn’t previously investigated. Therefore, the rationale of the present study was to compare irrigant activation techniques regarding debris removal from the anastomosis area located at different levels.

The null hypothesis of this study is that there is no significant difference in anastomosis cleanliness at different anastomosis levels between passive ultrasonic irrigation (PUI) using Irrisafe (Satelec, Acteon, France), sonic activation using EDDY, and the control group needle irrigation without activation (NA).

## Methodology

### Study design

All procedures performed were carried out in accordance with relevant guidelines, regulations and ethical standards of the ethical committee of research of the Faculty of Dentistry, Suez Canal University (approval number for the study: 137/2018.)

This study was a single-blinded study, conducted on 60 human mandibular first molar teeth extracted for periodontal reason, extracted teeth were collected from the outpatient clinics after approval of the patient. The sample size was calculated according to G*Power software version 3.1.9.2 at a significance level of *p* < 0.05. A minimum total sample size of 49 samples was sufficient to detect the effect size of 0.40, a power (1-β) of 80% at a significance level of *p* < 0.05 and partial eta squared of 0.14.

Teeth were rinsed under running water, then an ultrasonic scaler was used to remove calculus and soft tissue remnants from the root surface, then immersed in 5.25% NaOCl (Clorox, Egyptian Company for household cleaners, Cairo, Egypt) for 10 min for disinfection then stored in distilled water at room temperature till the time of the study. The experimental setup followed the technique described by Klyn et al. which used a copper cube based on the Bramante model [12] with the addition of a compressing component**.** It consisted of precisely fitted parts that were accurately assembled together using tightening copper screws. The tightening screws provided the necessary precision and compressive force to reposition the tooth sections and eliminate the 0.3 mm kerf space between sections and prevent their movement during instrumentation. Using the cube, a tooth can be sectioned and then reassembled recreating a complete root canal system and then disassembled again for evaluation. This allowed cut roots to serve as their own controls upon comparing anastomosis cleanliness before and after treatment. Roots were measured under the dental operating microscope (DOM) (Labomed; Labo America, Fremont, CA) and those with a length less than 12 mm were excluded. Teeth were decoronated and distal roots were removed. Roots with initial file size more than #15 were excluded to eliminate extreme difference in root dimensions between samples.

All roots were cut by a diamond disc (Isomet 4000 linear precision saw, Buehler, Lake Bluff, IL, USA) to establish a unified length of 12 mm for all roots for the purpose of standardization, to eliminate the variable of root canal length on the cleaning efficiency between groups.

Roots were scanned using cone beam computed tomography (CBCT) unit Scanora 3Dx (Kavo Dental, Germany) with exposure parameters; 90 kVp and 4 mA to detect the presence of a type V isthmus (complete anastomoses between both canals) at 2,4 and 6 mm from the apex and those with a partial connection or without an anastomosis or were replaced. Also, CBCT was also employed to exclude teeth internal or external root resorptions, calcified root canals, open root apices and to measure the angles of roots curvature using the Schneider technique [13], teeth with angles more than 20 degrees, internal resorptions or calcifications were replaced.

### Samples preparation

A #10 K-file (Dentsply-Maillefer, Ballaigues, Switzerland) was inserted into the root canals until it was visible under the DOM at the apical foramen to ensure the canal is patent. Length was taken, and 1 mm was subtracted to determine the established working length (WL). A glide path was established. The coronal openings were sealed with a cotton pellet and glass ionomer and the apical foramina were sealed by a double layer of nail polish to prevent their blockage by epoxy resin. The roots were sectioned at 2, 4, 6 mm from the apex—due to the higher prevalence of anastomoses in these levels- using an 0.3-mm-thick Isomet low-speed saw with a diamond blade. The blade was irrigated with IsocutPlus Fluid (Buehler, Lake Bluff, IL, USA) and water according to the manufacturer’s recommendations. The sections obtained were put into an ultrasonic bath with distilled water for 7 min to remove the debris originating from the cut. Pre-instrumentation images of the specimens (I_0_) were taken using a digital camera attached to a stereomicroscope (Carl Zeiss Stemi-2000, ZEISS, Germany) at 30 × magnification. Then, sections were reassembled in the metal cube and all external hex bolts were firmly tightened.

Hyflex CM rotary files (Coltène/Whaledent AG, Switzerland) were used to instrument all the root canals up to 35/0.04 taper. A total volume of 5 mL 5.25% NaOCl was used in each canal via a 30-gauge side-vented needle as 1 mL after each file. After complete instrumentation, the sections were disassembled, and images were taken (I_1_).

### Samples grouping

Sections were reassembled and divided into 3 groups according to the activation method. Samples were blindly and equally distributed among the groups.

#### Group 1 (NA)

1 mL 5.25% NaOCl was introduced into each root canal and left inserted 1 mm short of the WL in each canal and left without agitation for 30 s.

#### Group 2 (Irrisafe)

1 mL 5.25% NaOCl was introduced into each root canal and an Irrisafe tip (of a size 20/0.00 taper and length of 21 mm (IRR20)) was inserted to 1 mm from the working length, and activated for 30 s at a medium power setting using NSK Varios 370.

#### Group 3 (EDDY)

1 mL 5.25% NaOCl was introduced into each root canal for 30 s and an EDDY tip of a size (25/0.04 taper) attached to the airscaler (AS2000, NSK, Nakanishi, Tochigi-ken, Japan) was inserted to 1 mm from the working length to activate the irrigant for 30 s with 2 mm up & down amplitude in each root canal.

In all groups, irrigation was done using a 30-gauge side-vented needle (Steri irrigation tips 30 G, DiaDent Group International, Canada). All samples received a final rinse of 2 mL saline (1 mL in each root canal). Then canals were dried with paper points (Coltène/Whaledent AG, Switzerland). After final irrigation, post-activation images (I_2_) were taken. Images (I_1_, I_2_) were analyzed blindly using ImageJ software (National Institutes of Health, v1.51) to calculate the debris-filled area in the anastomosis before and after final irrigation. The outlines of anastomoses and the debris-filled areas were traced. Then the debris-free clean area was calculated by subtracting the debris from the total area. The anastomosis cleanliness percentage was calculated as follows:

$$\mathbf{Percentage}\boldsymbol\;\mathbf{of}\boldsymbol\;\mathbf{cleanliness}=\frac{Area\;free\;of\;debris}{Total\;area}\times100$$

To assess the improvement in anastomosis cleanliness after activation, the difference in percentage of cleanliness before and after activation was calculated by the following equation [14]:

$$\mathbf{Cleanliness}\boldsymbol\;\mathbf{difference}\boldsymbol\;\boldsymbol(\mathbf{CD}\boldsymbol)=Cleanliness\;percentage\;after\;activation\;(i2)-Cleanliness\;percentage\;after\;instrumentation\;(i1)$$

While Total isthmus cleanliness of each root was calculated as an average of the three levels (2, 4 and 6).

### Statistical analyses

Statistical analyses were done using the computer software SPSS for Mac OS version 26.0 (Statistical package for social Science, Armonk, NY, IBM Corp) at a significance level of 0.05. According to the Shapiro–Wilk normality test data were parametric (*p* > 0.05). Parametric test paired samples t-test were performed at 0.05 level followed by two-tailed significance test to evaluate CD before and after activation within the same group. However, to compare between different activation methods (NA, PUI, EDDY) and different levels (2, 4 and 6 mm) one-way analysis of variance (ANOVA) was applied. Post hoc tests were used to further compare between groups and levels in terms of Duncan’s multiple range tests (DMRTs).

## Results

### Intragroup comparison

Comparisons between mean percentage of isthmus cleanliness before and after different activation techniques (comparison within the same group): The mean of isthmus cleanliness (%) changed significantly from i1 (after chemomechanical preparation) to i2 (after final irrigation) at all measured levels (2 mm, 4 mm, and 6 mm) and as a total. Total isthmus cleanliness (%) changed significantly (sign. < 0.001*) from 54.72 to 60.15 in the final needle irrigation without activation (NA) group, from 53.95 to 79.42 in the Irrisafe group and from 42.22 to 76.53 in the EDDY group as revealed by paired samples t-test (Table 1).

**Table 1:** Comparison between mean % of anastomosis cleanliness before (i1) and after (i2) different activation techniques

Level	Anastomosis Cleanliness								
	**NA**			**Irrisafe**			**EDDY**		
	**i1**	**i2**	**Paired t-test**	**i1**	**i2**	**Paired t-test**	**i1**	**i2**	**Paired t-test**
			***p*-value**			***p*-value**			***p*-value**
**2**	**36.92** ± 3.07	**45.14** ± 3.15	< 0.001*	**58.65** ± 4.91	**81.13** ± 3.34	< 0.001*	**28.88** ± 5.87	**68.57** ± 6.25	< 0.001*
**4**	**59.29** ± 6.03	**64.59** ± 5.26	< 0.001*	**45.04** ± 4.99	**76.02** ± 4.37	< 0.001*	**44.29** ± 5.68	**82.19** ± 3.31	< 0.001*
**6**	**65.39** ± 7.02	**68.57** ± 6.96	< 0.001*	**58.75** ± 6.73	**81.36** ± 5.04	< 0.001*	**44.66** ± 4.47	**76.83** ± 6.16	< 0.001*
**Total**	**54.72** ± 3.66	**60.15** ± 3.40	< 0.001*	**53.95** ± 3.23	**79.42** ± 2.43	< 0.001*	**40.22** ± 3.15	**76.53** ± 3.09	< 0.001*

*significant at *p* < 0.05

Comparison of mean % of isthmus cleanliness between different levels (comparison within the same group): showed that the intragroup comparison showed that improvement in anastomosis cleanliness (i2-i1) in the needle irrigation without activation group (NA) was significantly higher in the apical 2 mm level compared to the 4 & 6 levels. The difference in cleanliness improvement in the Irrisafe and EDDY was insignificant between the three levels. (Table 1, Fig. 5).

### Intergroup comparison

**Fig. 1:**
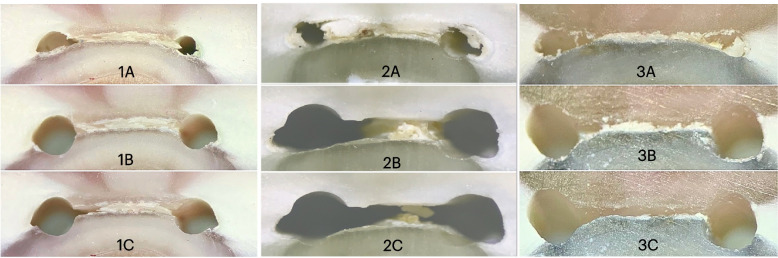
Digital images showing root sections at 2 mm from WL (**A**) before instrumentation (**B**) after instrumentation (**C**) after final irrigation with (1) NA, (2) PUI, and (3) EDDY. Note that (2C) shows irregularities created in the canal due to the ultrasonic tip coming in contact with the dentinal walls during activation (arrow)

**Fig. 2:**
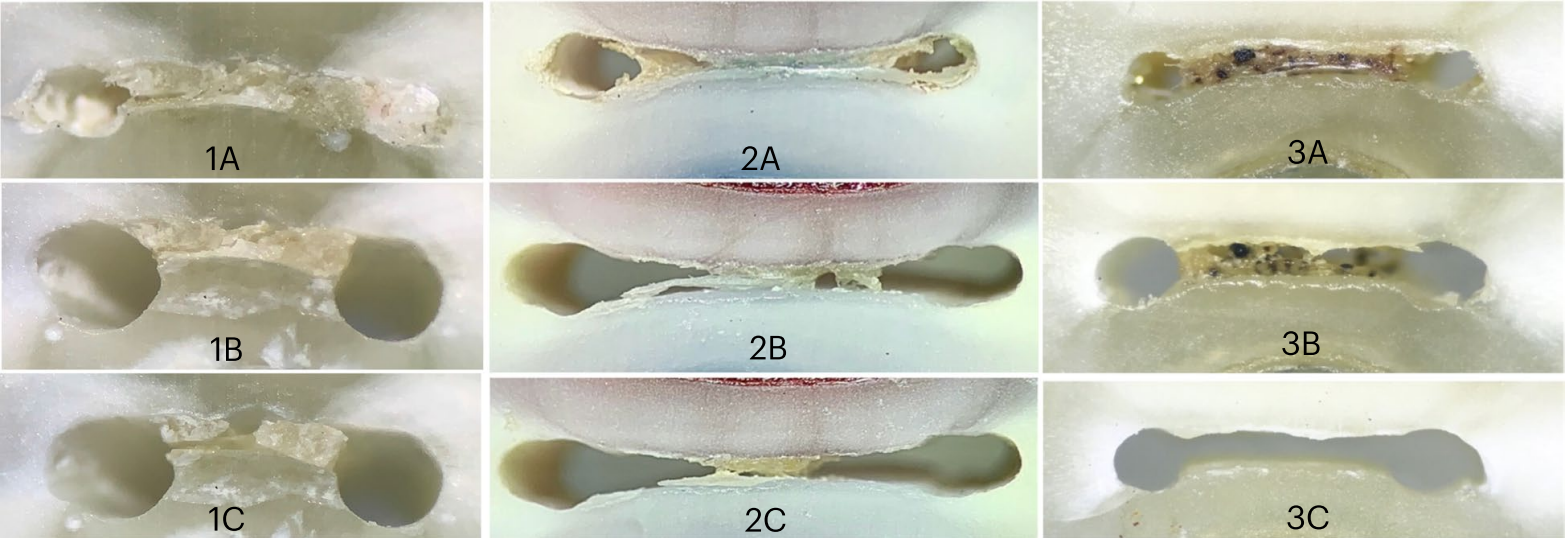
Digital images root sections at showing 4 mm from WL (**A**) before instrumentation (**B**) after instrumentation (**C**) after final irrigation with (1) NA, (2) PUI, and (3) EDDY

**Fig. 3:**
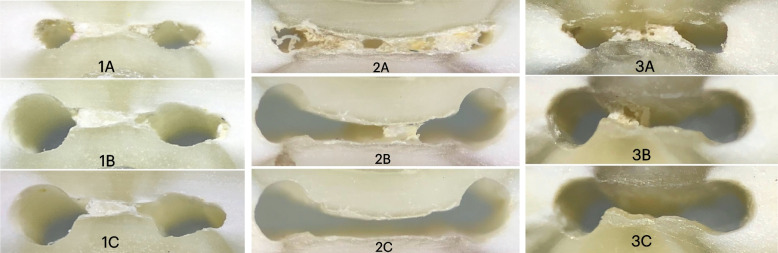
Digital images showing root sections at 6 mm from WL (**A**) before instrumentation (**B**) after instrumentation (**C**) after final irrigation with (1) NA, (2) PUI, and (3) EDDY

**Fig. 4:**
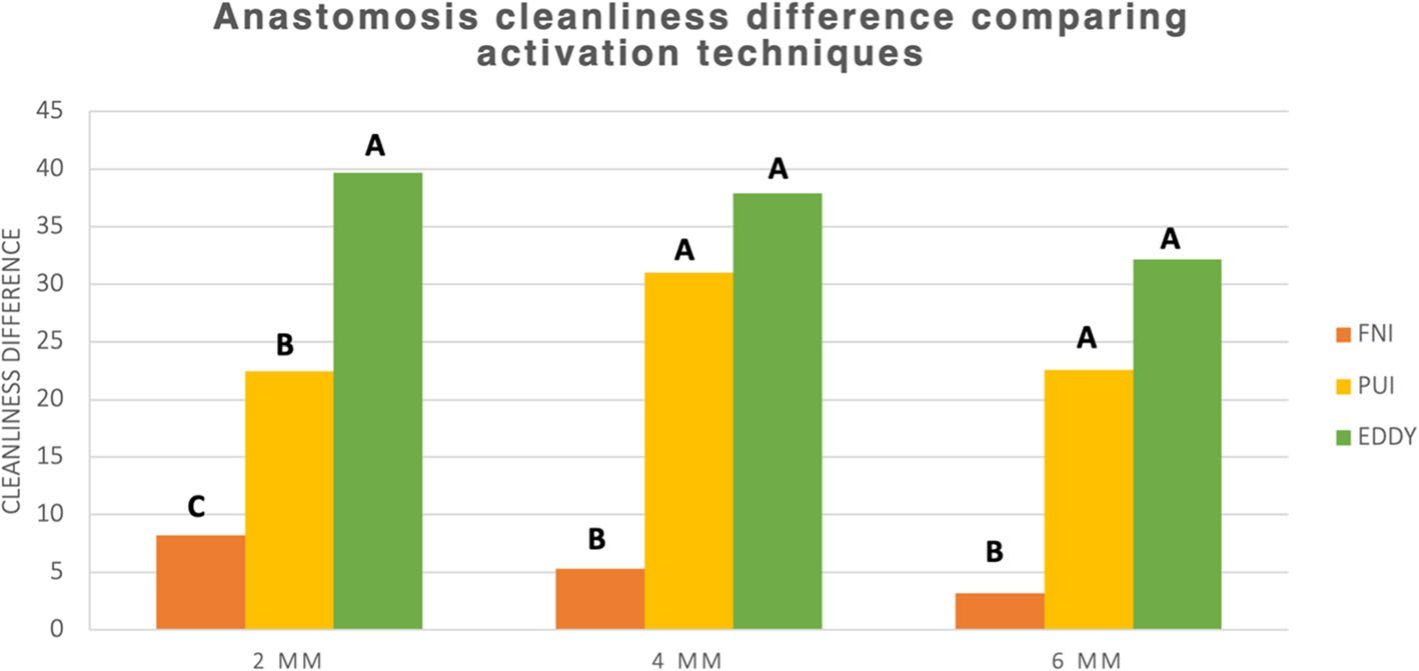
A bar chart comparing mean % of anastomosis cleanliness difference (cleanliness after instrumentation—after activation). **A** between groups **B** between root canal levels (different letters denote a significant difference)

**Fig. 5:**
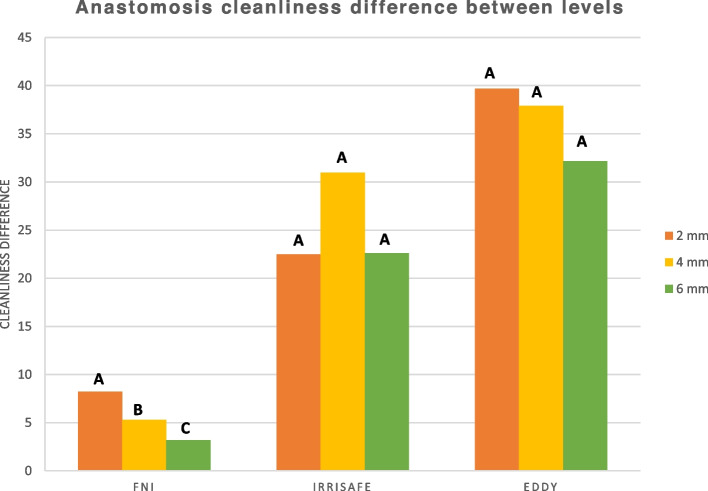
A bar chart comparing mean % of anastomosis cleanliness difference between levels (different letters denote a significant difference)s

Intergroup comparison revealed that EDDY and Irrisafe were significantly better than the control group at all levels and the difference between EDDY and Irrisafe was significant at 2 mm and insignificant at 4 and 6 mm (Table 2, Figs. 1, 2, 3, 4 and 5).

**Table 2:** Descriptive statistics of anastomosis cleanliness difference (Cleanliness percentage after final irrigation and activation– Cleanliness percentage after instrumentation.) comparing different activation methods at different levels (2 mm, 4 mm, and 6 mm) and as a total (average of the 3 levels). Data represented as mean ± SE (standard error). Differences were assessed by one-way ANOVA and Duncan post hoc tests at *p* < 0.05

Level	(CD) Anastomosis cleanliness difference			
	**B0**	**B1**	**B2**	***p*-value**
	**NA**	**Irrisafe**	**EDDY**	
**2**	**8.22 ± 1.23**^**cA**^	**22.48 ± 3.48**^**bA**^	**39.69 ± 5.69**^**aA**^	< 0.001*
**4**	**5.30 ± 1.18**^**bB**^	**30.98 ± 4.65**^**aA**^	**37.91 ± 5.79**^**aA**^	< 0.001*
**6**	**3.18 ± 0.33**^**bC**^	**22.61 ± 3.96**^**aA**^	**32.17 ± 2.92**^**aA**^	< 0.001*
**Total**	**5.43 ± 0.61**^**c**^	**25.48 ± 2.37**^**b**^	**36.31 ± 2.81**^**a**^	< 0.001*
***p*-value**	0.005*	**0.264 ns**	**0.817 ns**	

Means with different letters indicate significant difference. Small letter is for the comparison between different activation groups, while capital letters are for the comparison between different levels in the same group

^*^ significant at *p* < 0.05, ns; non-significant at *p* > 0.05 (NA: Final Needle Irrigation)

## Discussion

The fundamental goal of root canal therapy is disinfection of the root canal system followed by proper coronal and apical sealing, while the role of shaping is to remove infected dentin and provide space for irrigant penetration and subsequent obturation. Irrigation during shaping does not eliminate half of the debris that accumulates throughout instrumentation especially in the apical third [15]. Consequently, different agitation techniques have been recommended to enhance the efficiency and diffusibility of the irrigating solutions [16].

The copper cube allowed the investigation of root cross sections at the different stages of root canal preparation and irrigation so that each tooth served as its own control for a true analysis of the change in anastomosis cleanliness [2, 12, 17, 18]. Although the copper cube and sectioning method is older, and requires lots of steps, it reflects both soft and hard tissues debris while previously published studies that used micro-computed tomographic examination (micro-CT) measured only hard tissues debris[19].

The major objective of this study was comparing the cleaning efficacy of different irrigation activation techniques per se. Therefore, a simple irrigation protocol with only NaOCl was chosen to eliminate the added effect of any chelating agents as justified in several previous studies [11, 20–24] that focused solely on the difference between irrigation activation methods using only NaOCl. Furthermore, activation of EDTA could negatively affect dentin microhardness and results in root canal dentin erosion.

Roots were decoronated to standardize root length. This can be regarded as a technical limitation in this study because this allows easier access to the root canals, and there is no coronal reservoir of irrigation solution. Previous studies that focused on the prevalence of root canal isthmuses through different methodologies revealed high prevalence in mesiobuccal roots of maxillary molars and mesial roots of mandibular molars [25, 26]. Therefore, mesial roots of mandibular first molars were chosen for this study because it is the most common root to have anastomoses specially in the apical 6 mm [27]. Tissue debris tends to accumulate in the anastomoses during instrumentation which represent a major challenge for adequate cleaning and disinfection, which in turn impacts outcome of the root canal treatment particularly in infected root canals [28]. The Irrisafe tip was chosen because of its small size (0.2 with no taper) which is recommended for PUI in small canals such as the mesial canals of mandibular molars which were the subject of this study. The EDDY tip was chosen because it is operated at a higher frequency than the commonly used sonic devices like the EndoActivator. This higher frequency gives it a more powerful cleaning effect than the EndoActivator [29].

Samples were analyzed under a stereomicroscope at 30 × magnification because it allowed clear visualization of both root canals and anastomoses in the same frame, in contrast to other studies that employed scanning electron microscope in which only limited areas of the section could be scanned [11].

All the final irrigation techniques used in this study resulted in significant improvement in anastomosis cleanliness. As increasing the irrigant volume and activating it results in improved cleanliness [2, 14, 19, 29–32]. However, none of the techniques were able to fully clear the root canal system of debris.

The least improvement in anastomosis cleanliness occurred in the control group (NA) (Figs. 1, 2 and 3). This was in accordance with other investigations [30, 33]. At 2 mm, EDDY significantly improved cleaning compared to PUI (Figs. 1, 2 and 3). Therefore, the null hypothesis was rejected. This can be explained by the fact that the narrowest part of the root canal is the most apical part and that the streaming effect of ultrasonic tips decreases significantly when it touches the root canal walls, unlike sonic tips, which do not lose their effect upon contacting canal walls, rendering it more effective in cleaning the narrow 2 mm level [6]. Another explanation is that the soft polymer tip which is more flexible than ultrasonic permitted better introduction, and penetration and activation of the irrigant. Also, the displacement amplitude of EDDY (350 μm) is greater than that of PUI (75 μm). Furthermore, Swimberghe al 2019 [29] explained the superior cleaning performance of EDDY to the three-dimensional tip movement of EDDY in contrast with PUI, where the oscillation and the fluid stream occur mainly in one plane. Another difference between EDDY and Irrisafe is the up and down movement of the EDDY,while Irrisafe is kept steady. Contrarily, other studies found no significant difference at the 2 mm level between sonic activation and PUI [17, 34]. It is worth noting that they used EndoActivator as the sonic activation device, which produces less oscillation frequency with subsequent lower agitation capability than the airscaler that operates the EDDY tip, which can explain the superior cleaning of EDDY in the present study. Also, in those studies apical enlargement was done up to 40.04, while in this study it was 35.04, to create a more conservative preparation without compromising the biological objectives. Furthermore, mesial root canals of mandibular molars are small-sized canals, thus #35 is already larger than #30 master apical file which is the recommended size for the irrigant to reach the apical third [35]. This difference in the apical enlargement could have affected the ability of the PUI tip to vibrate freely thus producing these incompliant results [17, 34].

At the 4 mm and 6 mm levels, the difference between EDDY and PUI was insignificant. This can be attributed to the larger canal diameter at these levels, which allowed less-restricted vibration of both tips. This came in harmony with the previously published data comparing sonic to ultrasonic activation [18, 29, 34]. However, conflicting results were reported by Linden et al.[32], which could be attributed to the difference in methodology since they used micro-CT. Also, they admitted that debris moved from one canal to the other connected one which may have affected their results.

Regarding the intragroup analysis of NA, the 2 mm level showed the highest CD. This can be explained by the fact that after cleaning and shaping, this area is the one with the most packed residual debris [36]. So, even the slight addition of a non-activated irrigant as a final flush showed more improvement in this area compared to the wider 4 mm and 6 mm levels.

PUI at the 2 mm level was less effective than 4 mm but similar to 6 mm without significant difference. This can be attributed to the narrowness of this area may results in a more file-to-wall contact than the middle. Some of the samples even showed signs of canal deformation due to the ultrasonic tip touching the walls while vibrating (Fig. 1). The creation of irregularities from wall contact was supported by other studies [37, 38]. In the EDDY group, there was no significant difference between all levels. This can be explained by the fact that for the entire length of the tip, sonic energy generates just one single node and antinode. As a result, touching with dentinal walls has little effect on tip amplitude and hence tip movement [39].

It is important to emphasize that in this study, 1 mL of irrigant was activated for 30 s to investigate the exclusive effect of the activation technique. Increasing the irrigant volume, activation time, using a chelating agent and increasing the final apical enlargement size are considered limitations of the present study which may have different implications on the overall anastomosis cleanliness [11, 14].

## Conclusions

Under the limitations of the present study, we inferred that a final irrigation step is a must after root canal preparation to enhance anastomosis cleanliness. Sonic activation by EDDY promoted the best improvement in anastomosis cleanliness in the most apical part.

## Data Availability

This data made available on reasonable request to the corresponding author. Also,This research article is available as a preprint on research square (https://doi.org/10.21203/rs.3.rs-1854941/v1).

## References

[1] Nair PNR (2006). On the causes of persistent apical periodontitis: a review. Int Endod J.

[2] Thomas AR, Velmurugan N, Smita S, Jothilatha S (2014). Comparative Evaluation of Canal Isthmus Debridement Efficacy of Modified EndoVac Technique with Different Irrigation Systems. J Endod.

[3] Kabak Y, Abbott PV (2005). Prevalence of apical periodontitis and the quality of endodontic treatment in an adult Belarusian population. Int Endod J.

[4] Kirkevang L-L, Væth M, Hörsted-Bindslev P (2007). Risk factors for developing apical periodontitis in a general population. Int Endod J.

[5] van der Sluis LWM, Vogels MPJM, Verhaagen B (2010). Study on the Influence of Refreshment/Activation Cycles and Irrigants on Mechanical Cleaning Efficiency During Ultrasonic Activation of the Irrigant. J Endod.

[6] Plotino G, Grande NM, Mercade M (2019). Efficacy of sonic and ultrasonic irrigation devices in the removal of debris from canal irregularities in artificial root canals. J Appl Oral Sci.

[7] Siqueira Junior JF, Rôças IDN, Marceliano-Alves MF (2018). Unprepared root canal surface areas: causes, clinical implications, and therapeutic strategies. Braz Oral Res.

[8] Susin L, Liu Y, Yoon JC (2010). Canal and isthmus debridement efficacies of two irrigant agitation techniques in a closed system: Isthmus debridement in a closed canal system. Int Endod J.

[9] Fan B, Pan Y, Gao Y (2010). Three-dimensional Morphologic Analysis of Isthmuses in the Mesial Roots of Mandibular Molars. J Endod.

[10] Gu L-S, Ling J-Q, Huang X-Y (2009). A micro-computed tomographic study of the isthmus in the root canal system of mandibular first molar. Zhonghua Kou Qiang Yi Xue Za Zhi.

[11] Urban K, Donnermeyer D, Schäfer E, Bürklein S (2017). Canal cleanliness using different irrigation activation systems: a SEM evaluation. Clin Oral Investig.

[12] Bramante CM, Berbert A, Borges RP (1987). A methodology for evaluation of root canal instrumentation. J Endod.

[13] Schneider SW (1971). A comparison of canal preparations in straight and curved root canals. Oral Surg Oral Med Oral Pathol.

[14] Passalidou S, Calberson F, De Bruyne M (2018). Debris Removal from the Mesial Root Canal System of Mandibular Molars with Laser-activated Irrigation. J Endod.

[15] Paqué F, Boessler C, Zehnder M (2011). Accumulated hard tissue debris levels in mesial roots of mandibular molars after sequential irrigation steps: Debris reduction. Int Endod J.

[16] Weiger R (2021). Root canal irrigation: how much activation is necessary?. Dtsch Zahnärztl Z Int.

[17] Klyn SL, Kirkpatrick TC, Rutledge RE (2010). In Vitro Comparisons of Debris Removal of the EndoActivatorTM System, the F FileTM, Ultrasonic Irrigation, and NaOCl Irrigation Alone after Hand-rotary Instrumentation in Human Mandibular Molars. J Endod.

[18] Duque JA, Duarte MAH, Canali LCF (2017). Comparative Effectiveness of New Mechanical Irrigant Agitating Devices for Debris Removal from the Canal and Isthmus of Mesial Roots of Mandibular Molars. J Endod.

[19] Rödig T, Koberg C, Baxter S (2019). Micro-CT evaluation of sonically and ultrasonically activated irrigation on the removal of hard-tissue debris from isthmus-containing mesial root canal systems of mandibular molars. Int Endod J.

[20] Schäfer E, Vlassis M (2004). Comparative investigation of two rotary nickel-titanium instruments: ProTaper versus RaCe. Part 2. Cleaning effectiveness and shaping ability in severely curved root canals of extracted teeth. Int Endod J.

[21] Bürklein S, Schäfer E (2006). The influence of various automated devices on the shaping ability of Mtwo rotary nickel-titanium instruments. Int Endod J.

[22] Schafer E, Erler M, Dammaschke T (2006). Comparative study on the shaping ability and cleaning efficiency of rotary Mtwo instruments. Part 2. Cleaning effectiveness and shaping ability in severely curved root canals of extracted teeth. Int Endod J.

[23] Bürklein S, Hinschitza K, Dammaschke T, Schäfer E (2012). Shaping ability and cleaning effectiveness of two single-file systems in severely curved root canals of extracted teeth: Reciproc and WaveOne versus Mtwo and ProTaper: Single-file systems - shaping and cleaning. Int Endod J.

[24] Neuhaus KW, Liebi M, Stauffacher S (2016). Antibacterial Efficacy of a New Sonic Irrigation Device for Root Canal Disinfection. J Endod.

[25] Normanweller R, Niemczyk S, Kim S (1995). Incidence and position of the canal isthmus. Part 1. Mesiobuccal root of the maxillary first molar. J Endod.

[26] Estrela C, Rabelo LEG, de Souza JB (2015). Frequency of Root Canal Isthmi in Human Permanent Teeth Determined by Cone-beam Computed Tomography. J Endod.

[27] Paqué F, Laib A, Gautschi H, Zehnder M (2009). Hard-Tissue Debris Accumulation Analysis by High-Resolution Computed Tomography Scans. J Endod.

[28] Alves FRF, Andrade-Junior CV, Marceliano-Alves MF (2016). Adjunctive Steps for Disinfection of the Mandibular Molar Root Canal System: A Correlative Bacteriologic, Micro-Computed Tomography, and Cryopulverization Approach. J Endod.

[29] Swimberghe RCD, De Clercq A, De Moor RJG, Meire MA (2019). Efficacy of sonically, ultrasonically and laser-activated irrigation in removing a biofilm-mimicking hydrogel from an isthmus model. Int Endod J.

[30] Haupt F, Meinel M, Gunawardana A, Hülsmann M (2019). Effectiveness of different activated irrigation techniques on debris and smear layer removal from curved root canals: a SEM evaluation. Aust Endod J.

[31] Howard RK, Kirkpatrick TC, Rutledge RE, Yaccino JM (2011). Comparison of Debris Removal with Three Different Irrigation Techniques. J Endod.

[32] Linden D, Boone M, De Bruyne M (2020). Adjunctive Steps for the Removal of Hard Tissue Debris from the Anatomic Complexities of the Mesial Root Canal System of Mandibular Molars: A Micro-Computed Tomographic Study. J Endod.

[33] Iandolo A, Amato M, Abdellatif D (2021). Effect of different final irrigation protocols on pulp tissue dissolution from an isthmus model. Aust Endod J.

[34] Al Ahmari M, Al Maflehi N, Al Obaida M (2015). A comparison of the cleaning efficacy of ProRinse ® syringe needle, ProUltra ® PiezoFlow TM, and EndoActivator ® irrigation techniques using software program ImageJ. Saudi Endod J.

[35] Khademi A, Yazdizadeh M, Feizianfard M (2006). Determination of the Minimum Instrumentation Size for Penetration of Irrigants to the Apical Third of Root Canal Systems. J Endod.

[36] Yoo Y-J, Lee W, Kim H-C (2013). Multivariate analysis of the cleaning efficacy of different final irrigation techniques in the canal and isthmus of mandibular posterior teeth. Restor Dent Endod.

[37] Retsas A, Koursoumis A, Tzimpoulas N, Boutsioukis C (2016). Uncontrolled Removal of Dentin during In Vitro Ultrasonic Irrigant Activation in Curved Root Canals. J Endod.

[38] Boutsioukis C, Tzimpoulas N (2016). Uncontrolled Removal of Dentin during In Vitro Ultrasonic Irrigant Activation. J Endod.

[39] Ruddle CJ (2017). Endodontic Disinfection: The Sonic Advantage. Dent Today.

